# A Review of the Monitoring Techniques Used to Detect Oestrus in Sows

**DOI:** 10.3390/ani15030331

**Published:** 2025-01-24

**Authors:** Dannielle Glencorse, Christopher G. Grupen, Roslyn Bathgate

**Affiliations:** Sydney School of Veterinary Science, Faculty of Science, The University of Sydney, Sydney 2006, Australia; dannielle.glencorse@sydney.edu.au (D.G.); christopher.grupen@sydney.edu.au (C.G.G.)

**Keywords:** behaviour, cervical mucus, body temperature, electrical resistance, accelerometer

## Abstract

The agricultural industries have embraced the use of technologies as they improve efficiency and food security. The pork industry is no exception to this, as monitoring techniques and artificial intelligence allow for unprecedented capacity to track the physiological and behavioural condition of individual animals. This article reviews a range of those technologies in reference to the detection of oestrus in sows, a time when the ability to precisely ascertain physiological and behavioural changes associated with fluctuating hormone levels can have an immense impact on the economic profitability of the farm. The strengths and weaknesses of each technique from a practical application perspective are discussed, followed by considerations for further research and refinement.

## Simple Summary

In response to the need for improved efficiency, animal producers are increasingly incorporating novel technologies into standard husbandry procedures. One example where technology has the potential to enhance current practice is detection of oestrus in pigs. Correct identification of this time in the reproductive cycle will enable greater success rates of artificial insemination. This review outlines current knowledge of the oestrous cycle in the pig and standard procedures used for oestrus detection. It then goes on to discuss potential technologies that have been investigated in an attempt to improve these procedures, outlining the pros and cons of each method.

## 1. Introduction

An efficiently produced food supply is essential with the current rates of global population growth [1]. Intensively farmed animals such as pigs (*Sus scrofa)* have the capability for large-scale, sustainable production [2]. However, large herd sizes introduce challenges with animal management, predominantly due to the increased number of animals cared for by each stockperson [3]. The increase in herd size required to enable adequate supply leads to the need for new processes that are less labour-intensive and time-consuming [4].

One way to account for the increased labour demand from fewer staff is to introduce new tools, techniques, and technologies to quantify, objectify, and automate intensive farm practices [5]. An example of a production area that would benefit from more objective protocols is the management of breeding and reproduction. Sow reproduction is complex, as the process is affected by multiple factors, and results are variable within and between farms [6]. Conception rates vary depending on environment, management practices, animal factors, and stockperson skill and are therefore a process that would benefit from the application of novel techniques [7]. This is especially evident in the process of detecting oestrus and determining the appropriate timing of inseminations [8]. Detection of sexual receptivity, or oestrus, involves observation of behaviours, which dictate when sows are to be inseminated [9]. This is a subjective process that requires significant labour input and staff training to ensure accuracy [10]. This part of the breeding process is ideal for novel techniques, as improvement in the accuracy and precision of the current oestrus detection methods would enhance the overall productivity and profitability of pig farming [11]. However, the logistics of applying these technologies in industry are sometimes complex and should be considered in the development phase.

## 2. The Oestrous Cycle of Sows

**Figure 1:**
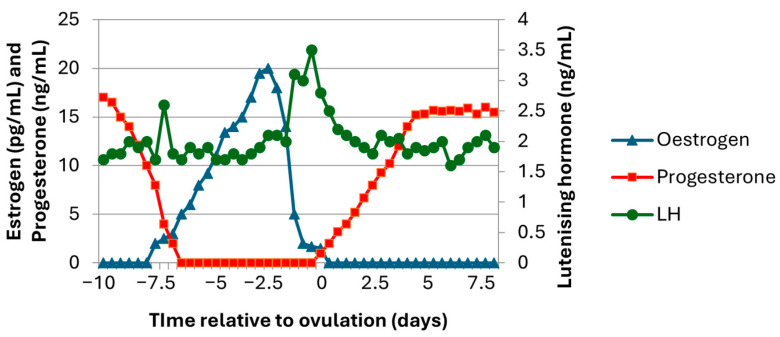
Representative schematic displaying the hormonal profiles of oestrogen, progesterone, and luteinising hormone that occur during the oestrous cycle of sows (adapted from [14]).

A basic understanding of the oestrous cycle in the sow is key for developing tools to detect physiological markers to aid in the detection of oestrus. The oestrous cycle of a sow is 18–21 days in length and comprises two distinct stages, namely, the follicular phase and the luteal phase [12,13]. The follicular phase is a period dominated by oestrogen where follicles develop in preparation for ovulation [14] (Figure 1). The luteal phase is the period where the reproductive tract prepares for a potential pregnancy under the influence of progesterone [15]. Domestic sows undergo polyoestrus cyclicity with continuous oestrous cycles throughout the entire year, regardless of season [16]. Detection of oestrus and prediction of when ovulation will occur within the period of oestrus are essential for successful inseminations.

### 2.1. Oestrus and Ovulation

Oestrus is a part of the follicular phase where sows display sexual receptivity [17]. Sows have an average oestrus length of 48–72 h, during which females display sexual receptivity behaviour and undergo physiological changes, particularly to the reproductive tract [8,18]. The length of oestrus varies among females with a reported hourly mean of 52.58 ± 8.56 and a range of 30–72 [19]. Sows undergo a gradual increase in sexual receptivity throughout oestrus, caused by secretion of oestrogen from the gonadotropin-dependent follicles on the ovaries [20]. During the mid to late stages of the follicular phase, the concentration of oestrogen increases and triggers a positive-feedback response from the hypothalamus [14]. This causes a surge in the release of gonadotrophin-releasing hormone (GnRH), triggering a subsequent surge of luteinising hormone (LH) from the anterior pituitary, which leads to ovulation 36–42 h after the initial GnRH release [21,22]. Thus, ovulation occurs approximately two-thirds of the way through oestrus and lasts for approximately 2–5 h, with multiple mature oocytes being released [23]. While ovulation timing occurs relatively consistently at 65–70% of the cycle across all sows [8,24], there is considerable variation in the length of oestrus, which means that the correct timing of ovulation can only be pinpointed retrospectively [25]. This means that there are no current procedures that can use this knowledge to enhance the accuracy of insemination timing [26]. As a result, variation in oestrus length is one of the main factors that have hindered producers from identifying the optimum insemination timing [21].

Oestrus length varies due to differing intervals between the onset of behavioural oestrus and ovulation, referred to as the oestrus-ovulation interval [17,27]. These intervals are dependent on several factors, including age and parity (with gilts having a significantly shorter period than multiparous sows [28,29,30]), season [31], and genotype [32]. As oestrus duration is relevant for determining when ovulation occurs and thus the optimum time for insemination, it would be beneficial to predict the duration of oestrus [17]. There has not been any observed correlation between the concentration of oestrogen and the length of observed behavioural oestrus [33,34]. This indicates that oestrogen has an impact on the changes to behaviour but does not support the hypothesis that the oestrus length is dictated by the concentration of oestrogen [18].

### 2.2. Physical Changes

The reproductive tract undergoes substantial physical changes under the influence of oestrogen [14]. The epithelial lining of the female reproductive tract contains receptors that respond to rising oestrogen concentrations in the early stages of oestrus [25,35] to create an environment that will support spermatozoa transit and survival during fertilisation [36]. This includes an increase in cervical mucus production and a change in its consistency [37]. Cervical mucus becomes thicker, denser, and smoother due to the presence of dissolved substances, such as sodium, chloride, and bicarbonate ions, and protein-based mucins [38]. Subsequently, there is a higher concentration of sodium in the mucus at ovulation [39]. Other changes include oedema and hyperaemia of the vulva and epithelial linings of the reproductive tract [25,35,40]. The uterine secretions provide lubrication and act as a medium for sperm transport, and contractions in the epithelial lining and reduced immune activity by the retention of leucocytes in subepithelial layers make the maternal tract environment conducive to sperm movement [41]. These physiological effects occur from the onset of the follicular phase until 2–3 days after the cessation of oestrus [25]. However, the intensity of each of these changes varies through the oestrus period [38]. Additionally, the magnitude of these physiological changes varies between sows as the concentration of oestrogen often differs from animal to animal [42].

### 2.3. Behavioural Changes

Sexual behaviour is controlled by the nervous system with neurotransmitter-based hormonal signals released from the hypothalamus [43]. Behaviours that occur during oestrus have several roles, such as attracting the boar and initiating sexual receptivity and copulation [18]. Three classifications of behaviours associated with oestrus have been described as attractive, proceptive, and receptive behaviours [18,44].

Attractive behaviours are often major [9]. These actions, which aim to attract the attention of potential mates, are repetitive and may involve other animals, with examples of posture changes, elevated activity levels, and vocalisation [45]. Proceptive or appetitive behaviours occur to induce sexual activity during proestrus and prior to standing heat [18,46]. During this time, sows will undergo behaviours such as female-to-female mounting, nudging, smelling, or licking the urogenital area of other sows; flank stimulation; pursuing other sows or boars; human interaction; and snout contact with other sows [14,18]. These behaviours become more frequent and apparent in response to visual, auditory, olfactory, and tactile stimuli from the male, and as such, producers use the physical presence of the boar to improve oestrus detection rates [44,47]. Receptive behaviours are those that demonstrate acceptance of copulation [48]. They occur when the sow undergoes mating and are usually determined by observing the presence of standing heat, an adjusted posture with immobility that enables the sow to accommodate the weight of the boar [8]. Standing heat, or standing oestrus, can be triggered by using the ‘back-pressure test’ to determine if the sow will display a rigid lordosis posture with the spine arching ventrally, squared limbs, and raised hips in response to pressure placed on her back [45]. The back-pressure test is the most commonly used method for detecting the receptivity of sows in commercial production, as the receptive behaviour occurs in response to physical stimulation by boars or stockpeople [18].

The timing of receptive behaviour is useful, as it occurs during the middle of the oestrus period and relatively close to ovulation [9,49]. However, the intensity of standing heat varies between individual sows, with some displaying minimal or no changes in posture, often making the process dependent on the ability and experience of stockpeople [50]. Some animals do not display any behavioural change while still undergoing the hormonal fluctuations that define oestrus [17,49]. Similarly, sows and gilts present varied oestrus behaviours, with the latter more likely to demonstrate clear boar-seeking and standing heat behaviours [51]. Extensive staff training is necessary to ensure that this behaviour is detected accurately [9].

## 3. Commercial Oestrus Detection Procedures

Oestrus detection is a multifactorial technique and, as such, requires more efficient tools to accurately predict the optimum time of insemination. An objective examination of the physical and behavioural changes that are present during oestrus could identify tools to improve the precision of oestrus detection [29].

Standard insemination protocols involve a double insemination occurring 12–24 h apart, beginning immediately after detection of standing heat [52]. Producers must be able to reliably determine the presence of oestrus and sexual receptivity in sows to ensure that insemination occurs close to the time of ovulation to facilitate high fertilisation rates [13]. This precision is necessary because the expected lifespan of liquid-stored spermatozoa in the female tract is up to 24 h, and the spermatozoa must transit the reproductive tract with sufficient time to fertilise the oocytes close to the time of ovulation [53,54]. Traditional commercial oestrus detection requires observation of behavioural and physiological changes [13] via visual assessment of sows once or twice daily [15].

### 3.1. Standing Heat

The major focus of oestrus detection protocols on farms is the standing heat test, which involves applying pressure to the back of the sow to identify lordosis [14]. The standing response can be detected by applying pressure to the back of the animal, but accuracy in detection of oestrus, independent of boar presence, is limited to 59% [45]. Undertaking this test in the presence of a boar increases the accuracy of detection to 90–100% [45]. This increase in accuracy is largely due to the pheromone 3α-androsterol present in boar urine, preputial fluid, and saliva, which induces sexual receptivity in sows and gilts [18]. There is also stimulation provided by visual and auditory pathways, but these are less well understood, despite their importance in accurate oestrus detection [55]. The presence of boars during oestrus detection and mating also results in oxytocin release from the pituitary of the sow, which increases the myometrial contractions that assist with the movement of spermatozoa through the reproductive tract [18,56]. This procedure is the most common method for oestrus detection in commercial production, but it is often utilised alongside several other methods to more accurately confirm oestrus [29].

### 3.2. Other Behaviours

In addition to detecting standing heat, producers will identify several other behaviours that are indicative of oestrus and initiated by the secretion of oestrogen [18]. These behaviours are often easier to detect if sows are housed in social groups and involve sow-to-sow interactions [57]. The behaviours observed by stockpeople include female-to-female mounting, snout contact, nose-to-urogenital interactions, flank stimulation, pursuing other sows, and approaching both humans and boars [9,29,56]. These behaviours are more difficult to identify in sows that are housed individually [58]. Other behaviours include increased vocalisation and increased activity levels, such as more walking and more frequent transitions between lying down and standing up [10,59]. While all of these behaviours are associated with oestrus, there is inherent variability between animals, and not all sows will display all of these signals [60,61]. To ensure that oestrus can be detected in these sows despite the lack of clear behaviour signals, alternate markers for the timing of insemination are required [62].

### 3.3. Vulval Swelling

Oestrus detection procedures can also include observation of physiological changes that occur as a result of increased oestrogen concentration [63,64]. Most notably, the vulva undergoes a series of changes, including swelling and reddening, because of increased blood flow to the reproductive tract tissues [65]. This is an obvious and simple way to detect change that occurs concurrently with behavioural oestrus [66]. This technique is often used in addition to the observation of behavioural signals [67]. However, the detection of these changes is also subjective and often neglected in the training of stockpeople [68]. Vulval swelling varies based on parity and is less evident in older sows that have undergone more parturition events [67], because each farrowing contributes to capillary damage in the tract and inhibits blood flow to the vulva during subsequent oestrus events [69].

### 3.4. Ear Flicking

The muscular contractions that occur within the uterine walls to facilitate sperm transit may be linked to the behaviour referred to as ear flicking [7]. These contractions are present during the standing heat response, which only occurs during early-to-mid oestrus [70]. It is hypothesised that these contractions could extend to the rest of the body, causing the ears to form a rigid position and quiver. This physical change, assumed to be involuntary, is only associated with oestrus and occurs alongside standing heat. However, ear flicking has not been examined in detail relative to ovulation [43] and likely varies depending on the configuration of the ear—upright or lop.

### 3.5. Decision-Making for Insemination Protocols

Oestrus detection through behavioural assessment will only successfully indicate the onset of the oestrus period but cannot predict the time of ovulation or the optimum time for insemination [29]. It is important to inseminate sows as close as possible to the time of ovulation, as this increases the rates of fertilisation and farrowing [71]. As discussed, it is difficult to predict the timing of ovulation within the oestrus period because of variations in individual oestrus length and behaviours displayed [43]. As ovulation cannot be successfully identified in real time, producers typically rely on oestrus detection once per day, followed by repeated inseminations every 24 h after the first-detected behavioural oestrus [72]. Conventional oestrus detection coupled with a double or triple insemination regime is used to increase the chances of fertilisation as it ensures that viable spermatozoa are present in the tract when the ova are ovulated [73]. Repeated inseminations are necessary if using liquid-stored spermatozoa, as the fertilising lifespan is greatly reduced 24 h post insemination [15,42,74]. However, this process is time-consuming, labour-intensive and wastes semen doses [74]. Productivity would be significantly improved by reliably predicting the timing of ovulation and thus an insemination time that achieves optimal fertility and fecundity with a single insemination [75].

## 4. Alternative Oestrus Detection Tools

The conventional method for detecting oestrus as described above is subjective, and hence the outcomes of inseminations that use this technique are often variable [7]. To improve on the efficiency of the current behavioural oestrus detection method, an accurate predictor of ovulation needs to be developed [76]. Any markers for predicting optimum insemination timing should be highly accurate with a low false-positive identification rate to be considered a valuable alternative to behaviour-based methods [77]. An ideal marker would involve a physical change that can be detected in the 24 h prior to ovulation to ensure that there is sufficient time available to plan and undertake insemination and ensure that spermatozoa are situated in the oviduct for fertilisation [78]. This means that a suitable biological marker would require a consistent, precise, and significant change in the 24 h period prior to ovulation. This marker also needs to be consistently present in a large majority of, if not all, sows [79]. The use of novel techniques and technologies that are automated, quantifiable, and objective would reduce labour requirements and increase the detectability of oestrus [80,81]. There are several alternative physiological and behavioural markers that may be exploited, including mucus composition, reproductive tract conductivity, oedema of the vulva, body temperature, and overall behaviour and activity levels [38,40,66,82]. These are discussed here, and a short summary of the pros and cons of each is presented in Table 1.

### 4.1. Saliva

Saliva has long been considered a good option for non-invasive analysis of animals for a variety of reasons [95]. Drawbacks to this technique include the variable volume of saliva produced by individuals under different conditions and at different times throughout the day [96], and therefore variation in concentration of molecules of interest. Additionally, saliva notoriously contains low concentrations of many molecules of interest [96] and so requires test methods with high sensitivity. Despite this, analysis of steroids in saliva is commonly reported and used in a diagnostic setting [97].

Successful detection of reproductive steroid concentrations in the saliva of pigs has been reported using gas chromatography and mass spectroscopy (GC-MS) [83]. These authors reported the ability to track ovarian activity via this method. Additionally, others have reported successful differentiation of protein profiles in sows at different stages of the oestrous cycle using MS [84]. While these reports give confidence about the presence of biomarkers that may flag oestrus, they are still far from a real-time, pen-side test that may be implemented in industry.

### 4.2. Cervical Mucus

Cervical mucus is a dense liquid produced within crypts in cervical tissue [25] consisting of a water-based solution containing dissolved ions such as sodium, phosphate, and chloride salts and proteins. Glycoproteins or mucins make up the insoluble component [85].

#### 4.2.1. Composition

The composition of mucus is often considered to be dynamic as it changes rapidly during oestrus [85]. Mucus is rarely homogenous, as the composition varies based on the location within the reproductive tract, with different molecules being produced and released in different sections [12]. In addition, the mucus composition is temporally variable with hormone concentrations dictating the quantity and structure of mucus, and the changes taking time to effect the entire mucus volume [98]. As the concentration of oestrogen steadily increases during oestrus, the mucosal glands regulate the quantities of the glycoprotein and ions accordingly [35,38].

Soluble components such as sodium chloride ions, bicarbonate, phosphates, and proteins are present in cervical mucus in addition to insoluble glycoproteins called mucins [38]. The composition of cervical mucus changes throughout the different stages of the oestrous cycle because of altered concentrations of soluble ions and proteins [35,38,99]. During pro-oestrus and early oestrus, the main components of secreted mucus are glycoproteins, which predominantly consist of sialic acid [100,101]. This is significant for the movement of spermatozoa, which is reduced due to the dense and highly viscous nature of these molecules [35]. Proteomic investigation of cervical mucus across the oestrous cycle has also identified changes in the proteomic profile of mucus with one particular protein, identified as dimethylarginine dimethylaminohydrolase 2 (DDAH2), being present at a >3-fold higher concentration in the days following weaning compared with the time around oestrous [102]. However, no further publications have investigated this promising discovery. The increasing oestrogen concentration in late oestrus leads to a more watery consistency of mucus [103], providing a medium for sperm transit following insemination [38,104]. This is observed by pig producers as an accumulation of mucus on the vulva and is associated with the onset of behavioural oestrus [37].

#### 4.2.2. Volume

In addition to the modified composition, oestrogen also causes an elevated volume of mucus within the reproductive tract, leading to hydration of the epithelial tissue [25,105]. This is due to an increase in the quantity of active mucus-secreting cells found in the epithelium of the vagina that occurs during pro-oestrus and oestrus [106].

#### 4.2.3. Crystallisation

Crystallisation, otherwise referred to as arborisation, is a visual phenomenon that involves the formation of patterns in air-dried samples of cervical mucus [35]. This has been observed in saliva and cervical mucus in several species, including humans [107], dogs [108], cattle [35,106], and pigs [37,38]. The phenomenon is caused by sodium chloride and phosphate salts within the mucus accumulating around the insoluble mucins [37,99,106]. These patterns change in response to the concentration of the salts in the mucus, with higher concentrations causing the formation of crystalline, fern-like patterns observable under a light or compound microscope [37,38].

Detection of fern-like crystallisation patterns in cervical mucus has been associated with the oestrus period in sows and cows [22,25,37]. Due to the relationship between crystallisation and elevated oestrogen levels, it has been suggested that the stage of oestrus and optimal insemination time could be determined by monitoring mucus samples [79,106].

Zink and Diehl (1984) [86] demonstrated that the concentration of electrolytes increases prior to the onset of behavioural oestrus signs. More recently, the highest proportion of sodium chloride has been found to occur simultaneously with the peak of circulating oestrogen; therefore, obvious ferning patterns would be expected to occur predominantly during the follicular phase and, in particular, during oestrus [38,109]. This explains the absence of mucus ferning during the luteal phase and pregnancy, as the high progesterone levels are negatively correlated with oestrogen levels [25,110].

Several studies have identified crystallisation patterns present at the onset of behavioural oestrus, with the signal changes occurring 24–48 h prior to ovulation [38,62,106]. Despite these results, a clear and simple crystallisation classification protocol has not been developed or tested in commercial settings, as the technique is still somewhat subjective, with different observers providing varied or opposing assessments of a sample [79]. This alternative oestrus detection tool could be implemented on-farm with minimal training and equipment requirements if a quantifiable and universally identifiable set of guidelines can be established. One potential obstacle for this technology is the labour required to assess mucus samples from each sow. For this reason, an important addition to research in this area would be the development of artificial intelligence and machine learning to accurately, reliably, and rapidly interpret samples at the pen side.

### 4.3. Vaginal Electrical Resistance (ER)

Electrical resistance (ER) is an electrical measurement that has been used to detect the changes in mucus composition that occur throughout the different stages of the oestrous cycle [111]. Mucosal tissues conduct an electrical charge, which can be measured by introducing a current into the epithelial lining [86]. This charge is a result of the presence of an increased concentration of sodium and chloride ions, which provides a greater intensity of ions through which electrical currents can be conducted [79,112]. As the vaginal epithelium undergoes hydration, an increase in the electrolyte content in mucus causes an elevation in the electrical conductivity of the tissue 48 h prior to behavioural oestrus, which can be detected by certain ER technologies [25,86].

The observed fluctuations in electrical potential during the oestrous cycle show a peak ER (measured in ohms) during the luteal phase, indicating that elevated progesterone severely reduces mucus production and conductivity [25,79,113]. Most sows recorded basal resistance levels 1–2 days before obvious behavioural oestrus signs occurred [114]. The preovulatory LH surge occurs 6.2 ± 4.5 h after a subsequent increase in mucosal ER [40]. While this suggests a potential marker for predicting the time of ovulation, the variability between sows indicates that this may not be a precise method [114]. Further research needs to be undertaken to ensure that the precise time of ovulation can be reliably predicted using ER [115], as there are several factors that affect the accuracy of ER measuring devices. These variances and the time required to test each sow, on top of hygiene and biosecurity concerns around the reuse of the ER probe, currently prevent this technology from industry adoption.

#### 4.3.1. Predicting Insemination Timing

Several studies have examined the use of vaginal ER probes to determine the optimal time for insemination in pigs. In the Okinawan native Agu pig, in which oestrus signals are difficult to visually observe, vaginal ER monitoring was found to improve the scheduling of artificial inseminations [41]. In crossbred sows, natural matings and artificial inseminations performed after a substantial increase in vaginal mucosal conductivity resulted in conception and farrowing rates that were comparable to those achieved using conventional behavioural detection of oestrus to time the inseminations [79,86,105]. Also, in a recent comparison of liquid-stored and frozen-thawed semen inseminations, similar conception and farrowing rates were obtained when changes in vaginal ER values were used to inform the timing of insemination [116].

Despite the comparable farrowing rates obtained using vaginal ER probes for detecting the optimum time for insemination, this technology has not been implemented in commercial practice, primarily due to the lack of improvement in farrowing rates [40,116,117]. In addition, the collection of data from ER probes requires collation of daily readings to ensure that the decreased ER value is identified, and biosecurity is of concern when sharing equipment between animals [113]. However, the use of vaginal ER measuring devices warrants further investigation, as they have the potential to quantify previously subjective and inconsistent techniques and overcome variation in insemination efficiencies [114].

#### 4.3.2. Probe Location and Use

Electrical resistance values fluctuate between different regions within the female reproductive tract [118]. The resistance is higher when measured within 10 centimetres of the vulva and progressively decreases as readings are taken closer to the cervix [79,105,119]. Similarly, vestibular and vaginal resistance have contrasting changes during the oestrous cycle [120]. During pro-oestrus, vaginal resistance decreases while vestibular resistance increases [86,105,116]. While the reason for the contrasting changes is unknown, the practical application of this information is that consistency of probe placement is of the utmost importance to ensure results are valid [40,111]. Different-sized sows may have significant differences in the length of the reproductive tract, which could introduce variation that prevents accurate measurement of ER [121]. It is crucial that there is consistent and full contact between the probe and the tissue to obtain accurate readings [117].

#### 4.3.3. Parity

The parity of the sow affects the vaginal ER reading. Sows with parities greater than two have higher resistance readings than sows that have yielded a single litter, as older animals tend to have more damage to the epithelial walls, resulting in reduced permeability of circulating oestrogen into the cells [76]. In addition, older sows are usually larger in size and have longer reproductive tracts, which can impede connection between the tissue and probe at the required location [122]. As a result, the vaginal ER changes indicative of oestrus onset are less pronounced in older sows and therefore may impede accurate prediction of ovulation timing [117].

#### 4.3.4. Operator Error

Stockpeople must be competent in using ER probes, particularly due to the importance of probe location and the contact point for obtaining accurate ER measurements [111]. Variation is also expected to occur between ER probes with differing specifications as well as in commercial settings where various operators use the same equipment [119]. These issues highlight the need for training to ensure the correct use of equipment to measure ER accurately [25,86,118,123].

### 4.4. Oedema of the Vulva

With the rapid development of camera systems and artificial intelligence, novel means of detecting oestrus in sows have been trialled. Early investigation of technology to monitor this biological feature of oestrus has shown promising results, with light detection and ranging (LiDAR) cameras being able to accurately detect an increase in vulvar size that was correlated to behavioural oestrus [124]. While this technology may not be suitable as a sole decision-making tool, it could be used to reduce labour by flagging sows that require manual checking or used in combination with other technologies, such as monitoring changes in body temperature or classification of changes in cervical mucus to allow for fully automated detection of the physiological changes associated with oestrus. More recently, camera footage was combined with machine learning as a sole means of oestrus detection with high levels of accuracy [125,126]. This provides exciting potential for the future of precise and objective detection of oestrus with an associated decrease in labour costs. The practical use of cameras and machine learning for assessing vulva size in group-housed sows should be investigated, as well as the use of the technology in sows without docked tails, as these could be logistical issues in welfare-friendly production systems.

### 4.5. Body Temperature

Body temperature can be used for monitoring physiological changes to normal bodily functions [127]. Temperature monitoring in sows has previously allowed detection of farrowing, disease, and oestrus [102].

The fluctuations in body temperature vary based on the location of the reading on the body [69]. Radio-telemetric devices implanted under the skin are highly accurate and collect continuous data. They have been used to detect an increase in mean ear temperature 6–12 h before oestrus onset in cattle [127]. However, subdermal measurement of temperature is more variable than rectal and tympanic areas, as superficial tissues are influenced by ambient environmental conditions [128]. An alternative non-invasive thermometer that requires minimal training to use correctly is an infrared thermometer [129,130,131]. This technology uses a handheld pointer device that obtains accurate temperatures by utilising infrared waves to measure below the surface of the skin [132]. This process is simple, quick, repeatable, and hygienic and also minimises sow distress by preventing the need for physical contact [66]. While infrared technology is the most accessible method for temperature monitoring, the use of more advanced thermometers can reduce the interference from ambient temperature and improve the accuracy of this oestrus detection method [102].

Thermal technology can be used to measure temperature across an entire surface instead of being limited to a single point when using an infrared thermometer [69]. This process involves imaging the body location and using software to calculate the average temperature [133]. This provides a greater ability to accurately detect the true temperature of the location without environmental effects [87,134] and may enable the detection of oestrus-specific temperature changes that could be used as a marker for insemination timing [130]. It also has the potential to be used for identifying other physiological events such as parturition and disease [102,135]. Early detection of these factors can improve production efficiency if stockpeople can be made aware of individual animals that require directed treatment or assistance [127]. To identify the effectiveness of body temperature monitoring in a commercial setting, the results obtained using both simple, handheld infrared thermometer devices and a more advanced automated thermal system should be compared. This technology has become commercially available in recent years [88], and its performance should be closely monitored to help inform further refinement.

Automated detection of changes to baseline body temperature can be detected using video-based thermography [131]. The main benefit of using video-detected temperature is that it can collect continuous data at much smaller time intervals than a handheld device [136]. In addition, handheld devices require a consistent technique, significant animal contact, and are more laborious to use [66]. Automated thermometer recording can identify individuals that have shown a specified temperature requiring action or intervention [137]. As a result, there are minimal labour requirements, as stockpeople are notified to enable action [80]. However, the presence of faeces on or near the vulva can affect the recorded temperature and should be considered when developing this technology [59].

Ear temperature has been measured at the base of the ear in sows [127]. Similarly, body temperature is often measured in the rectum, as this location correlates closely with core body temperature [135]. Another potential location for observing body temperature is the vulva. During oestrus, there is an increase in blood flow to the area resulting in hyperaemia, and as blood infiltrates the surrounding capillaries, an elevated vulval temperature is observed [130,134]. These fluctuations in vulva temperature have been observed in cows [138,139] and sows [134]. Peak vulval temperature was detected between 12 and 36 h prior to ovulation in sows using a digital infrared thermometer [66]. In addition, vulva temperature was found to decrease significantly (1.14 °C) from baseline, 12 h prior to ovulation [102]. These changes have been attributed to the elevated concentration of oestrogen, which causes swelling and associated temperature changes to the reproductive tract [140]. While this is useful information, the reason for the decrease in temperature prior to ovulation is unclear. The use of vulva temperature changes has been observed during oestrus, but current research has not investigated fluctuations in other body locations. Using temperature fluctuations in more obvious body locations as a signal for oestrus could be beneficial to allow for herd-level monitoring of group-housed sows when visual access to a faeces-free vulva is hampered.

### 4.6. Quantification of Behaviour

Behaviours change to represent the physiological or reproductive status of an animal [141]. For example, nest-building behaviour is used to signal the onset of parturition [142]; reduced activity levels indicate illness or disease that could be linked with a variety of conditions such as lameness [143]; a lower feed intake can signal bacterial infections [144]; and an increase in activity level signals the presence of sexual behaviour [58,145]. Changes in behaviour are often the first visible sign to indicate issues with sickness [141], stress [146], feeding patterns [147], social and hierarchical changes [148], parturition and birth [149], and oestrus [150]. Improved efficiency in livestock production is possible by identifying and classifying these behaviours using large-scale data collection precision technologies [151]. In order for these technologies to be able to detect biological and physiological events, an understanding of the movement and behaviour associated with these events must be defined.

As the average ratio of animal:stockperson increases, there is a corresponding decrease in animal welfare, an issue of particular importance with growing trends in group housing of sows [82]. Direct observation of animals is required to identify stress, pain, disease, discomfort, feed intake, reproductive capacity, and growth. By limiting the observation time per animal, changes in these parameters can go unnoticed [152]. By detecting and reporting on behaviour discrepancies using automated technologies, compromised growth can be detected early, and long-term repercussions can be prevented [153]. The large herd size of commercial pig farms has encouraged the use of precision technology to improve labour allocation, maintain animal welfare standards, and increase production efficiency [127,136]. Several technologies have been developed for automated, precision monitoring of domesticated and production animals. There are a range of uses for precision technologies in relation to reproductive performance [154].

#### 4.6.1. Time-Lapse Video

Time-lapse video footage has been used to identify the frequency and duration of boar visitations by sows, whereby 95% of sows demonstrated standing heat and signalled with sufficient time for a mating to be completed [50]. However, in this study of 50 animals, 42% of the sows had a false-positive oestrus event detected on one or more occasions, which demonstrates low specificity [50]. This technology has been refined to allow more direct, quantifiable measures of boar visitation and oestrus length [155]. Collar-based transponders were used with nearby receivers to detect and record the frequency and duration of visits to a boar. Peak visitation occurred 2–3 days after the first observed increase in the frequency of visits [156]. While this is useful information for breeding management, it is usually only obtainable retrospectively. Electronic sow feeder stations, time-lapse video footage, and transponder data are largely obtained retrospectively and therefore are incapable of real-time oestrus detection [123]. In addition, these technologies rely on sows having access to the boar at all times [10]. Full boar contact for 24 h per day is absent from most commercial systems, particularly as it leads to habituation and less overt behaviours being expressed [76].

Further to this study is the potential to combine video footage with artificial intelligence to detect changes in sow behaviour that may indicate oestrus. Initial investigation of one of these systems has enabled detection of oestrus with an accuracy of 86% [89], while a different system incorporated into a commercial farming practice improved the reproductive outcomes compared with conventional subjective assessment of oestrus detection [90]. While improvements must be made to improve the efficiency of this method, particularly to enable it to operate with minimal labour, it shows promise for enabling objective oestrus detection.

#### 4.6.2. Electronic Oestrus Detection Station

Electronic identification of sows is necessary in welfare-friendly production systems, particularly in group housing, outdoor housing, and reduced confinement sow pens [47]. Using collar-based transponders to monitor the visits to an electronic oestrus detection (EED) station located adjacent to a boar pen, Blair et al. [47] found a correlation between conventionally detected behavioural oestrus and EED-based boar visitation lengths [47]. Despite the correlation between boar visitation and conventional oestrus, the incentive for visitation to the EED station may be based on habituation to feeding systems that these sows were previously exposed to, indicating the need for further exploration [157]. Using a form of electronic oestrus monitoring system may improve detection when compared to conventional behavioural detection, resulting in a reduction in the number of non-productive days per sow [158]. Future studies should identify the relationship between hormonal fluctuations and boar visitation during oestrus. This would determine if there is a correlation that could be manipulated as a predictor of the correct time for insemination.

This concept has been taken further with the development of a ‘bionic boar’ [91]. These authors developed a robotic model boar that mimics the smell, sound, and touch of a real boar to detect oestrus in sows. Combined with machine learning, they proposed that the ‘bionic boar’ could accurately predict sows displaying behavioural oestrus [91]. There are several advantages to this technology in the removal of boars from farms (reducing cost, risk to stockpeople, space requirements for boars, and welfare concerns for boars housed as teasers). However, the cost of this technology may be a limiting factor to its use, along with its capacity to detect oestrus in group-housed animals.

#### 4.6.3. Pedometers

Motion-based technologies, such as pedometers, can provide a step count and accumulative motion index based on internal algorithms of acceleration magnitude [159]. Sows often display heightened activity levels and increased levels of interaction with other sows during oestrus [45]. This technology would be particularly useful for detecting increased activity levels in individual sows that do not display obvious behavioural oestrus [80,160,161,162]. The use of pedometers has been successful for monitoring lameness in cattle [163,164], but the mode of attachment is important [159,165]. Sensors placed on legs can differentiate between body and leg movements, whereas sensors placed on the head or collar cannot [156,166,167]. However, the attachment of sensors to sows is difficult in locations such as the ear, leg, or face due to potential interference with normal behaviours and the motions the system is attempting to detect [168]. In addition, the retention rate of sensors is reduced in sows that are group housed or when they are insecurely attached [150].

#### 4.6.4. Accelerometers

Accelerometers are small devices that can give quantifiable signal profiles for physical activity [169]. The hardware within an accelerometer converts movement-based oscillations into electrical signals [165]. An acceleration force produces a voltage output that is amplified and converted into a digital value that is corrected for the effects of gravity [141]. This technology has been applied in several species to quantify the orientation of an object and the change in positioning over time that occurs as a result of movement. This has allowed profiles of several actions and motions to be defined, including human motion [170,171,172], feeding behaviour in dairy cows [173], postsurgery behaviour in dogs [174], feeding behaviour in sheep [151], and illness in cats [175]. Single-axis accelerometers have been used to successfully record and distinguish specific biting and chewing actions in dairy cattle and sheep [165,176]. The detection of rumination action allows for monitoring of typical feed consumption behaviour and identification of health issues that interfere with normal feeding activity. However, the use of these accelerometers is often limited as they can only detect one plane of movement.

Three-dimensional accelerometer technologies can detect posture and movement with highly frequent measurements [159]. Tri-axial accelerometers use three planes of movement, namely, vertical swing, medio-lateral, and cranio-caudal movement [82]. Accelerometers attached to sows for a continuous length of time have been used to form unique signal profiles of activity on three planes for several activities, namely, walking, feeding, rooting, and lying [92]. Walking was detected by a single profile showing forward acceleration, while lying sternally and laterally was identified by a reduction in the vertical acceleration and a single-sided horizontal biased acceleration, respectively [92]. Despite the success of differentiating between these movements and postures, walking and rooting behaviours are often difficult to separate [5]. Differentiation of these behaviours would be beneficial, with walking being indicative of correct motion and the absence of lameness and rooting indicative of healthy feeding patterns [82]. To identify more complex motion events, a cumulative activity level can be used instead of relying on a unique signal profile [151].

Sensors can overcome the time-consuming, labour-intensive nature of continuous behavioural observation, a task that is often not feasible in animal production, particularly in large herds [165,177]. To ensure that these behaviours can be identified and classified by accelerometers in sows, further studies are required to quantify the acceleration involved in each activity of interest and to enable the development of automated systems for notifying stockpeople. While accelerometers allow identification and quantification of behaviours that exploit the information that is readily available, the efficacy of accelerometers is affected by a range of factors.

##### Correct Prediction

One aim of accelerometer technologies is to identify a signal that can be used to predict the associated behaviour [178,179]. However, the signal that is obtained is not always capable of predicting the presence of a behaviour with 100% certainty [10,137]. The correlation between accelerometer signals and observed activity often yields high percentages of true positive predictions, but this is usually accompanied by a portion of false positives and false negatives [151]. These false predictions are often a result of individual variation in behaviour expression and could be caused by factors such as disease, weight, social interactions, and differences in temperament [151].

##### Weight

There are differences in signal profiles from accelerometers based on variation in the weight of individuals [141,180]. This is believed to occur because an individual with a larger mass can produce a greater force, which increases the intensity of a movement [181]. This needs to be taken into account when developing technologies for automated detection of movement of individual animals.

##### Gait

Gait is the pattern of movements an animal is capable of forming to allow motion [182]. This range of movements is often highly variable in humans and monkeys due to their bipedal locomotion [146]. Even simple movements such as walking and standing are often erratic, unbalanced, and asynchronous and can be different based on the individual involved [183]. In contrast, movement in quadruped species is more consistent and usually based on simple, repetitive patterns with a full range of uninhibited movements [141]. When this information is considered, the accuracy of accelerometer-based behaviour prediction is higher in quadrupeds [141].

##### Attachment Site

Optimising the attachment site of the sensor to the animal is important due to the need to ensure that data are collected continuously and effectively [184]. Attachment of any electronic system needs to be secure to ensure continuous data collection and to prevent injury from consumption or contact with the transponders involved [185]. Successful attachment in some species is not always straightforward and is substantially more difficult in social and wildlife species [165]. Sensors attached to the horn, nose, and forehead of cows using medical tape have been used to determine the location that can most accurately detect rumination behaviours [165]. The accelerometer attached to the horn was most accurate, detecting 99% of observed activity [165]. In sows, ear tag-attached accelerometers have been used to detect lameness in group-housed animals [186] and determine the onset of farrowing [187]. However, ear-based attachment can introduce variation in the movement detected by the accelerometer [188]. In addition, differences in ear size and conformation between breeds and individuals could introduce discrepancies in classifying movements [189].

Collar-based accelerometers have been used successfully for classifying and predicting normal behaviour in domestic cats [141], sheep [151,181], dairy cattle [81], and individually housed sows [92]. However, the use of collars in group-housed sow systems is difficult, as shown by a study where 44 occasions of removal by the pigs occurred within a group of 40 gilts [47]. The proposed reason for these difficulties was based on differences in attachment and adjustment strategies used by individual personnel as well as the social interactions and temperament of the sows [93,190].

Recently, several versions of ear tag style accelerometers for oestrus detection in sows have been released [191,192], demonstrating that this technology is practical and attractive to producers.

#### 4.6.5. Auditory Analysis

An alternative technology that has recently been investigated for monitoring a variety of states in pigs is that of auditory analysis. This technique involves the recording of vocalisations made by pigs in different states (e.g., in oestrus) and training artificial intelligence to filter and recognise patterns of sound related to that particular state [94]. The difficulty with this technology is the inherent background noise of a commercial piggery that must be filtered out to enable recognition of individual pig vocalisation.

## 5. Conclusions

Commercial AI protocols using liquid-stored and frozen-thawed boar spermatozoa require accurate oestrus detection to ensure that high conception rates occur within a herd. Identification of sows in oestrus is currently a highly subjective activity, and the timing of ovulation relative to the onset of oestrus is variable among individual animals. Consequently, multiple inseminations are needed to allow for inaccurate identification of oestrus onset and the subsequent unknown time of ovulation within the period of oestrus. As the global demand for food continues to grow, an additional issue is the concurrent increase in average herd size with larger animal-to-stockperson ratios. This highlights the need to develop simpler, faster, and more accurate oestrus detection protocols that fit within existing commercial operations. While emerging technologies show potential for detecting oestrus and predicting the time of ovulation with a high level of accuracy, improvements are required before many of these are suitable for widespread adoption by industry.

## Figures and Tables

**Table 1:** Summary of technologies assessed in this review for improved oestrus detection in sows.

Technology	Benefits	Disadvantages	References
Biomarkers in saliva	Minimally invasive	Not currently possible in real time	[83,84]
Biomarkers in cervical mucus	Minimally invasive	Can be difficult to collect samples; not currently possible in real time	[85]
Mucus crystallisation	Minimally invasive, low tech	Currently subjective and time-consuming	[37,38]
Electrical resistance	Already commercially available, non-invasive	Variable success rates, potential biosecurity threat	[25,86]
Body temperature	Can be performed remotely; low cost	Currently not suitable for group-housed sows	[87,88]
Behavioural analysis—video	Can be performed remotely; incorporation with machine learning would remove labour requirements	Technology not yet developed to be sufficiently accurate; high setup costs	[89,90]
Behavioural analysis—electronic detection	Can be performed remotely	High setup costs; variable accuracy reported in some systems	[47,91]
Behavioural analysis—pedometers and accelerometers	Can be performed remotely, already commercially available	High setup costs; ear tag attachment not always secure	[92,93]
Auditory analysis	Can be performed remotely	Managing background interference in a noisy environment; not suitable for group-housed sows	[94]

## Data Availability

No new data were created or analysed in this study. Data sharing is not applicable to this article.
